# Development of nomogram based on immune-related gene FGFR4 for advanced non-small cell lung cancer patients with sensitivity to immune checkpoint inhibitors

**DOI:** 10.1186/s12967-020-02679-0

**Published:** 2021-01-06

**Authors:** Li Wang, Zhixuan Ren, Bentong Yu, Jian Tang

**Affiliations:** 1 Precision Medicine Center of Oncology, The Affiliated Hospital of Qingdao University, Qingdao University, Qingdao, 266003 China; 2 Department of Oncology, TCM-Integrated Hospital of Southern Medical University, Southern Medical University, Guangzhou, 510000 China; 3 Department of Thoracic Surgery, The First Affiliated Hospital of Nanchang University, Nanchang, 330000 China

**Keywords:** NSCLC, FGFR4, Immune checkpoint inhibitors, Immune-related genes, Nomogram

## Abstract

**Introduction:**

Immune checkpoint inhibitors (ICIs) have become a frontier in the field of clinical technology for advanced non-small cell lung cancer (NSCLC). Currently, the predictive biomarker of ICIs mainly including the expression of PD-L1, TMB, TIICs, MMR and MSI-H. However, there are no official biomarkers to guide the treatment of ICIs and to determine the prognosis. Therefore, it is essential to explore a systematic nomogram to predict the prognosis of ICIs treatment in NSCLC

**Methods:**

In this work, we obtained gene expression and clinical data of NSCLC patients from the TCGA database. Immune-related genes (IRGs) were downloaded from the ImmPort database. The detailed clinical annotation and response data of 240 advanced NSCLC patients who received ICIs treatment were obtained from the cBioPortal for Cancer Genomics. Kaplan–Meier survival analysis was used to perform survival analyses, and selected clinical variables to develop a novel nomogram. The prognostic significance of FGFR4 was validated by another cohort in cBioPortal for Cancer Genomics.

**Results:**

3% of the NSCLC patients harbored FGFR4 mutations. The mutation of FGFR4 were confirmed to be associated with PD-L1, and TMB. Patients harbored FGFR4 mutations were found to have a better prolonged progression-free survival (PFS) to ICIs treatment (FGFR4: P = 0.0209). Here, we built and verified a novel nomogram to predict the prognosis of ICIs treatment for NSCLC patients.

**Conclusion:**

Our results showed that FGFR4 could serve as novel biomarkers to predict the prognosis of ICIs treatment of advanced NSCLC. Our systematic prognostic nomogram showed a great potential to predict the prognosis of ICIs for advanced NSCLC patients.

## Introduction

Lung cancer is the most common cancers with high mortality. Lung cancer was responsible for an estimated 2,093,876 new cases and approximately 1,761,007 deaths in 2018 alone [1]. Lung cancer was mainly divided into two types: non-small-cell lung cancer (NSCLC) and small cell lung cancer (SCLC). Among them, NSCLC accounts for more than 85% of lung cancer. Traditional treatment of NSCLC mainly including surgery, radiotherapy, chemotherapy and targeted therapy. However, only about 20% of patients with locally early NSCLC (stage I and II) have chance to a radical resection [2]. Moreover, the mutations of tumor-driving genes such as EGFR, BRAF, ERBB2 and rearrangement of ALK or ROS1 only exist in less than half of NSCLC patients, which suitable for receiving targeted therapy [3]. For advanced NSCLC patients who cannot be treated with surgery or targeted drugs, platinum-based chemotherapy has been used as a standard treatment [4].

Nowadays, immune checkpoint inhibitors (ICIs) have proven to be a major breakthrough achievement in cancer treatment in the past decade. Especially, ICIs have shown convincing efficacy and tolerable safety in monotherapies or combined treatments in NSCLC [5, 6]. However, the objective response rate of ICIs is only about 17%, indicating that it could benefit only a limited subpopulation of patients [7]. Therefore, it is the greatest challenges to be able to predict the efficacy of ICIs treatment and find a suitable population during clinical application. At present, there are some predictive biomarkers have been found to be related to ICIs, such as programmed death-ligand 1 (PD-L1) expression, tumor mutational burden (TMB), tumor-infiltrating immune cells (TIICs), mismatch repair (MMR) and microsatellite instability-high (MSI-H) [8–11]. However, Gainor et al. [12] found that NSCLC patients with EGFR mutations and ALK rearrangements might low response from ICIs treatment. There are still lack of clear biomarkers that can predict the response of ICIs treatment. Shen et al. [13] suggested that PD-L1 expression status alone is insufficient in determining which patients should be offered ICIs therapy. Due to the heterogeneity and high somatic mutation rate of NSCLC cells, multivariate analyses of the sensitivity to ICIs involving the multiple biomarkers and the genomic signatures confirmation of patients are necessary. Therefore, it is essential to explore a systematic nomogram to predict the prognosis of ICIs treatment in NSCLC.

In order to provide novel biomarkers to improve clinical diagnosis, evaluate the immune status of patients, and evaluate of the sensitivity of ICIs treatment, the differential expression of immune-related genes in NSCLC tissue samples should be studied. In this work, IRGs were downloaded from the ImmPort database [14]. We conducted an extensive analysis based on transcript and clinical data obtained from the TCGA databases. In addition, IMPACT sequencing of 240 NSCLC tumor/normal pairs treated at MSKCC with ICIs therapy obtained from the cBioPortal for Cancer Genomics. In order to further explore the potential relationship between IRGs and clinicopathological information, we developed a systematic nomogram to predict the prognosis of NSCLC with ICIs treatment.

## Methods

### Data download and processing

We analyzed the differential expression of IRGs between tumor and adjacent normal tissues. A total of 2499 IRGs were downloaded from the Immunology Database and Analysis Portal (ImmPort) [14]. The clinical annotation and response data of 240 advanced NSCLC patients who received ICIs treatment and were obtained from the cBioPortal for Cancer Genomics [15]. The involved patients with advanced NSCLC were treated with ICIs monotherapy (pembrolizumab) or combination (pembrolizumab + ipilimumab) and profiled by targeted next-generation sequencing (NGS). The clinical information of this cohort including age, sex, cancer type, progress free survival (PFS), and smoking history. The assessment of TMB was conducted in the published clinical trial [15]. The efficacy of ICIs treatment was assessed by Response Evaluation Criteria in Solid Tumors (RECIST) version 1.1. And PFS was assessed as the time that patients remained responsive to ICIs treatment. A total of Eighty-four tumor tissue were sent for PD-L1 expression assessment.

### Comprehensive analyses of IRGs

We conducted differentially expressed analysis on the IRGs between NSCLC and adjacent normal tissues using the “Limma”, with thresholds of false discovery rate (FDR) < 0.05 and a log2 |fold change| > 2.0 in TCGA cohort. Additional RNA-Seq profiling tool “edgeR” to verify consensus degree with Limma. Then, Gene ontology (GO) and The Kyoto Encyclopedia of Genes and Genomes (KEGG) enrichment analyses were performed using the clusterProfiler package, with thresholds of P and FDR values less than 0.05, indicating statistical significance.

### Development of nomogram

The differentially expressed IRGs were analyzed in cBioPortal for Cancer Genomics. The Kaplan–Meier survival analysis was used for univariate analyses of the cBioPortal cohort, and the logrank tests were used to detect significant differences. In addition, Kaplan–Meier Plotter online tool was used to perform survival analyses for all NSCLC patients [16]. A nomogram based on variables with selected by the univariate analyses. The “rms” package was used to construct the nomogram. C-index and Calibration plot were conducted to estimate accuracy and discriminative value of prognostic nomogram. The exploration of the relationships between key IRGs and six subtypes of tumor-infiltrating immune cells (TIICs) by TIMER online tool [17].

### Verification of the prognostic significance of IRGs

External validation of the IRGs was performed by another cohort [TMB and Immunotherapy (MSKCC, Nat Genet 2019)] in cBioPortal for Cancer Genomics [18]. This cohort analyzed the clinical and genomic data of 1662 advanced cancer patients treated with ICI, and 5371 non-ICI-treated patients, whose tumors underwent targeted next-generation sequencing.

### Statistical analyses

All statistical analyses were performed using the GraphPad Prism software 7.0 and R software 3.6.1, and Student’s t tests were used to determine statistical significance. All significant comparisons were defined as P < 0.05.

## Results

### Differentially expressed IRGs

**Fig. 1:**
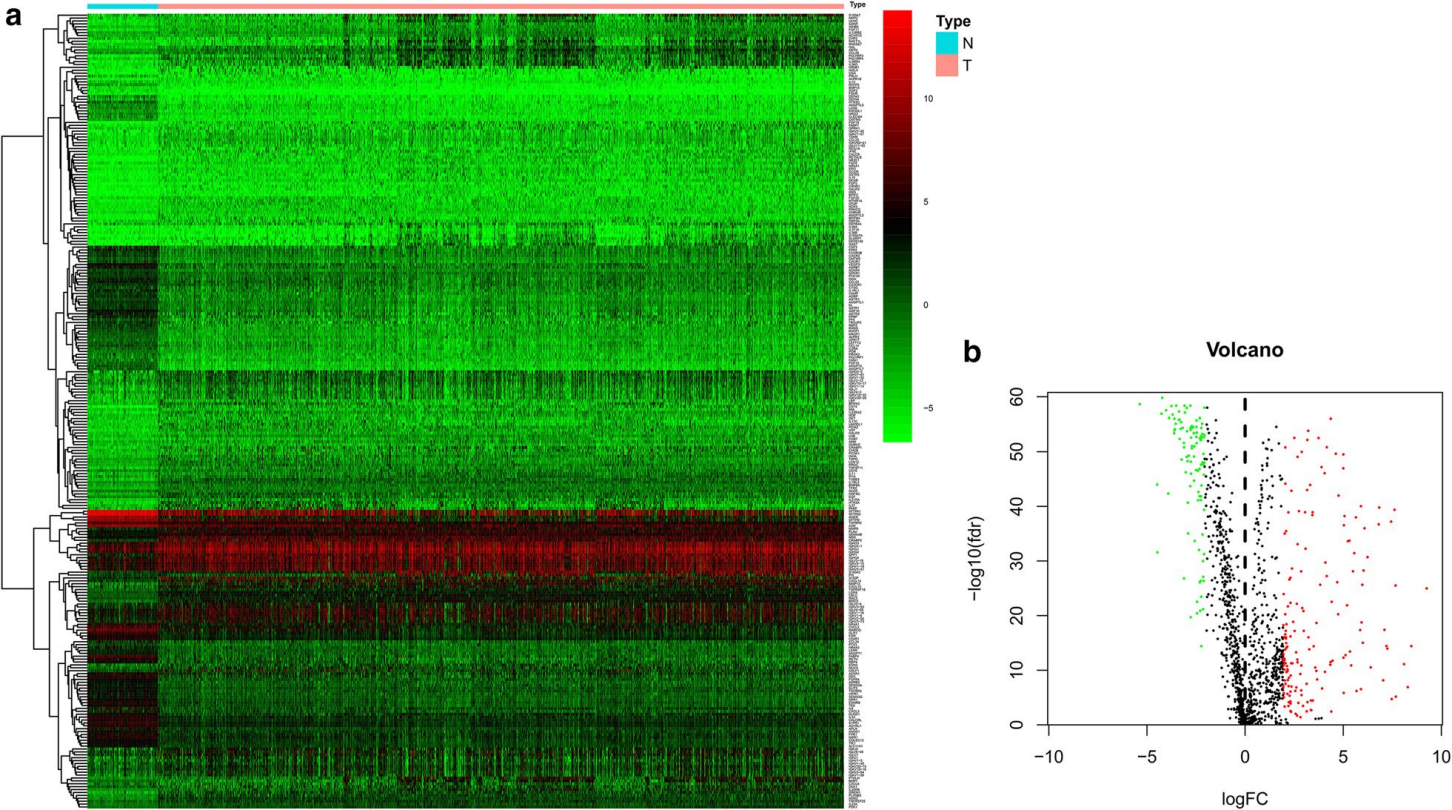
Differentially expressed IRGs between NSCLC and normal tissues. **a** Heatmap of differentially expressed immune-related genes. **b** volcano plot of differentially expressed immune-related genes. Red, up-regulated IRGs, green, down-regulated IRGs

In TCGA cohort, 274 differentially expressed IRGs were identified, including 170 up-regulated and 104 down-regulated genes with the cutoff point of false discovery rate (FDR) < 0.05 and a log2 |fold change| > 2.0 (Fig. 1a, b). Differentially expressed IRGs are shown in Additional file 1. And the results of additional RNA-Seq profiling tool “edgeR” are shown in Additional file 2.

### GO and KEGG enrichment analyses

**Fig. 2:**
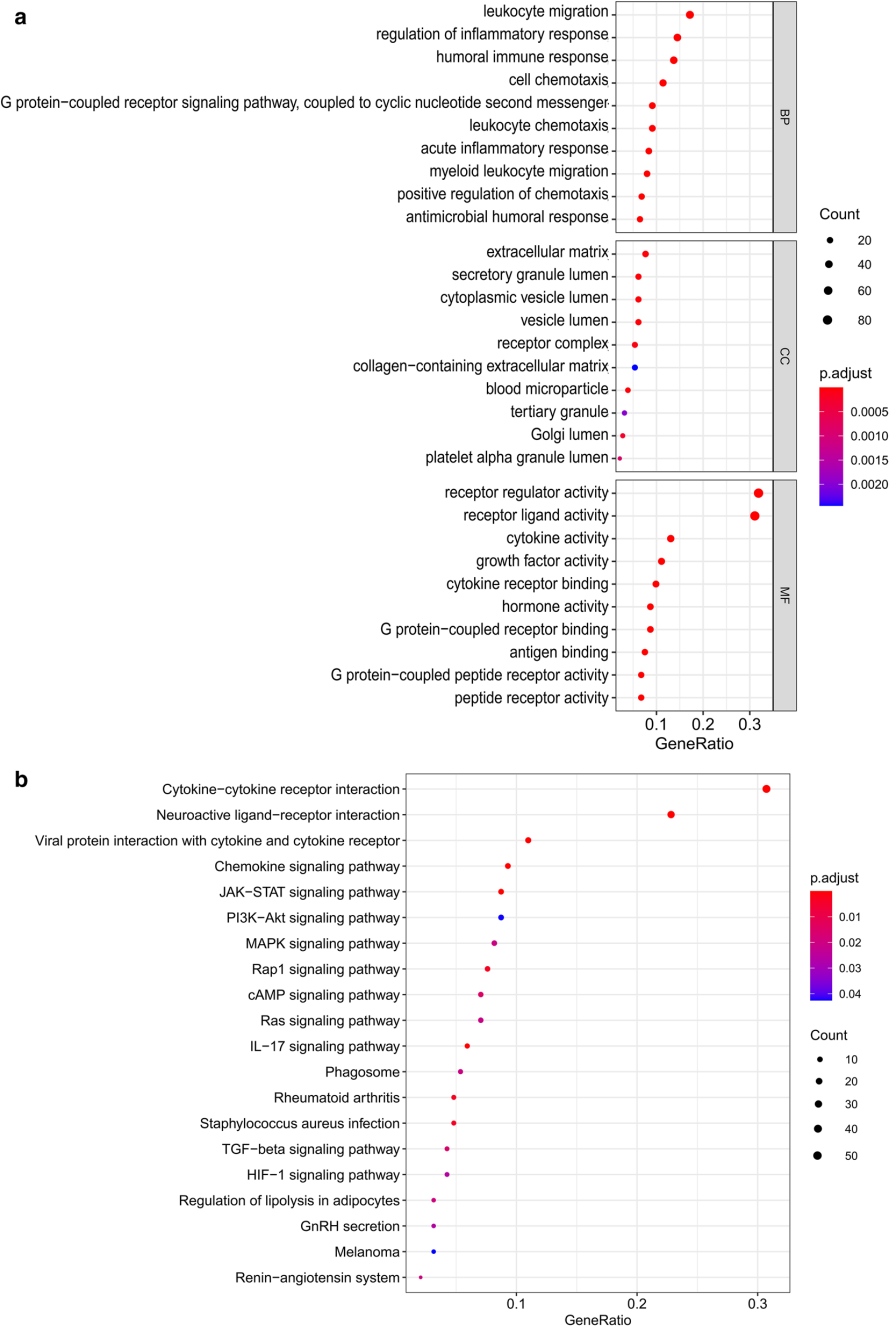
Go and KEGG analysis of differentially expressed IRGs. **a** Bubble plot of enriched GO terms. **b** Bubble plot of KEGG

GO enrichment analysis showed that the differentially expressed IRGs were mainly associated with the BP terms, including leukocyte migration, regulation of inflammatory response, humoral immune response and cell chemotaxis. In addition, the CC terms showed that the IRGs were associated with extracellular matrix, secretory granule lumen, and cytoplasmic vesicle lumen. Moreover, the MF terms were mainly included receptor regulator activity, receptor ligand activity, and cytokine activity (Fig. 2a). KEGG enrichment analysis showed that these differently expressed IRGs were mostly enriched in cytokine–cytokine receptor interaction, neuroactive ligand–receptor interaction, and viral protein interaction with cytokine and cytokine receptor (Fig. 2b).

### Differentially expressed of IRGs analyses by cBioPortal for Cancer Genomics

**Fig. 3:**
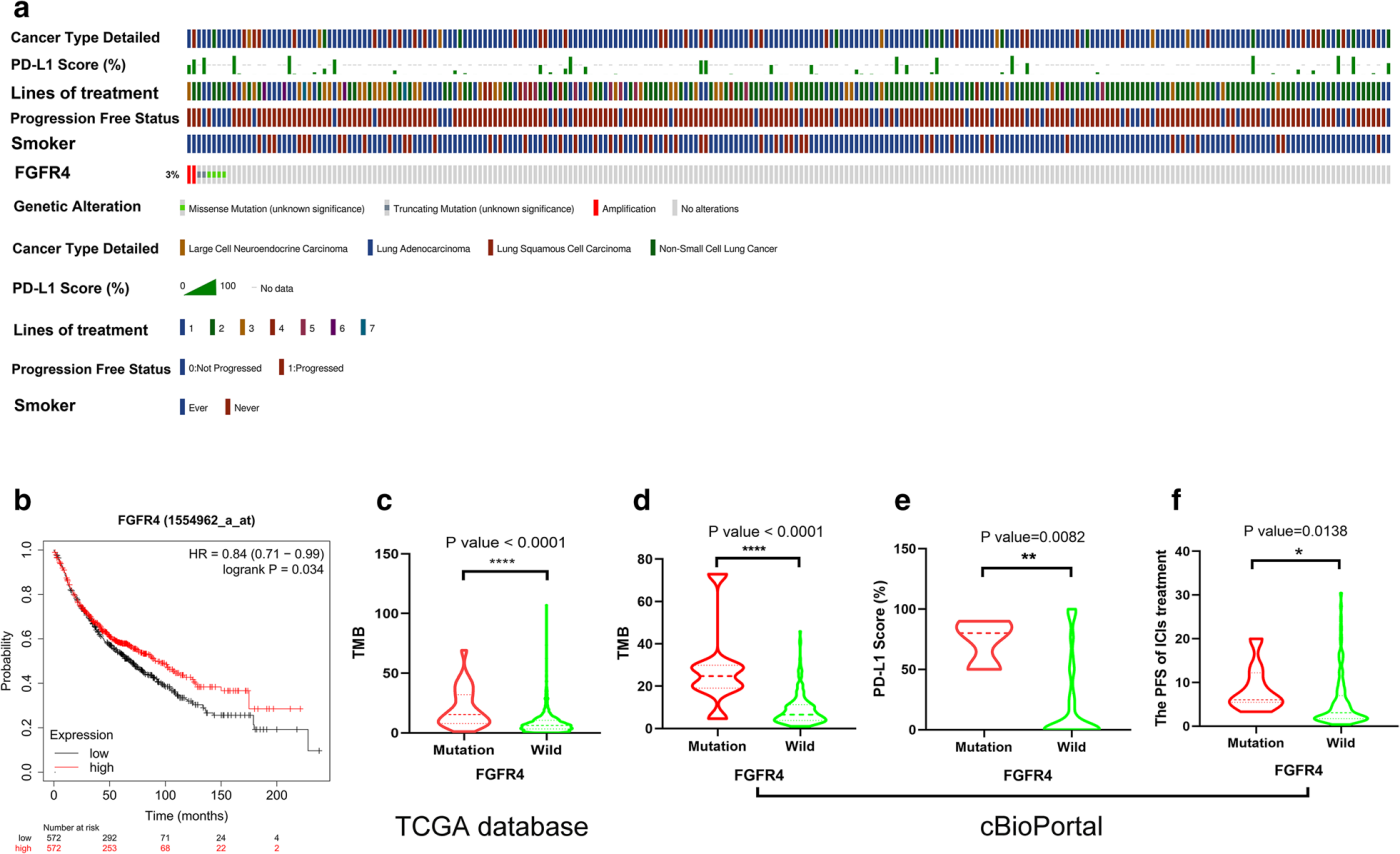
The mutation status of FGFR4 is closely associated with the prognosis of NSCLC. **a** The prevalence of FGFR4 mutations in NSCLC patients based on the cBioPortal for Cancer Genomics; **b** the expression of FGFR4 was closely related to the prognosis of lung cancer patients; **c** the mutation status of FGFR4 is associated with the TMB level in TCGA cohort; **d** the mutation status of FGFR4 is associated with the TMB level in cBioPortal for Cancer Genomics; **e** the mutation status of FGFR4 is associated with the PD-L1 expression in cBioPortal for Cancer Genomics; **f** the mutation status of FGFR4 is associated with the PFS of ICIs treatment in cBioPortal for Cancer Genomics

Total of 274 differentially expressed IRGs were analyzed by cBioPortal for Cancer Genomics. Only FGFR4 was associated with the prognosis of NSCLC patients with ICIs treatment. In addition, the mutation frequencies of FGFR4 was 3% in NSCLC (Fig. 3a). Then, we further investigated the relationship between the survival of NSCLC patients and the expression of the FGFR4 protein. Kaplan–Meier Plotter online tool showed that the expression of FGFR4 was closely related to the prognosis of lung cancer patients (HR = 0.84 [0.71 − 0.99], log‐rank P = 0.034) (Fig. 3b). In addition, the mutation status of FGFR4 is associated with the TMB level, PD-L1 expression and the PFS of ICIs treatment (Fig. 3c, d). FGFR4 mutations were associated with a higher TMB value (P < 0.0001) in TCGA cohort. Moreover, FGFR4 mutations were associated with a higher TMB value (P < 0.0001) and a higher PD-L1 score (P = 0.0082) in advanced NSCLC patients in cBioPortal.

### Construction of nomogram

**Fig. 4:**
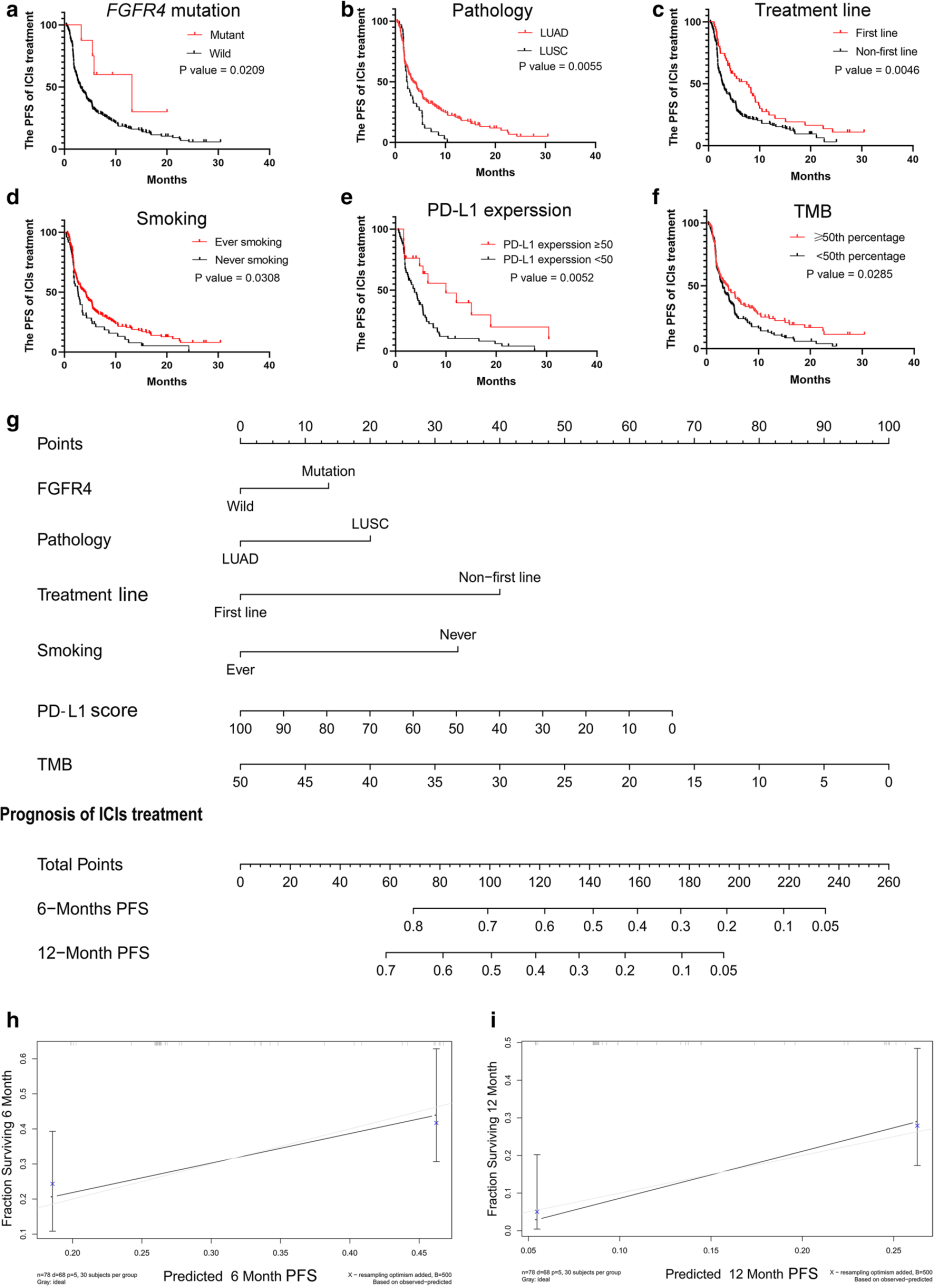
Novel nomogram to predict the prognosis of advanced NSCLC with ICIs treatment. **a** The survival curves for ICIs treatment based on the mutational status of FGFR4; **b** the survival curves for ICIs treatment based on the pathology; **c** the survival curves for ICIs treatment based on treatment line; **d** the survival curves for ICIs treatment based on the smoking; **e** the survival curves for ICIs treatment based on PD-L1 expression; **f** the survival curves for ICIs treatment based on TMB; **g** the novel nomogram based on these clinical information to predict the prognosis of ICI treatment; **h** the calibration plot for the 6 month PFS of nomogram; **i** the calibration plot for the 12 month PFS of nomogram

Total of seventy-nine NSCLC patients of the cBioPortal for Cancer Genomics with integrated information on clinical information, targeted sequencing and PD-L1 expression were involved in the construction of the systematic nomogram. We determine the variables of nomogram by performed univariate analyses. There are multiple variables were confirmed to be significantly associated with the prognosis of NSCLC with ICI treatment including FGFR4 mutation (P = 0.0209), pathology (P = 0.0055), treatment lines (P = 0.0046), smoking (P = 0.0308), PD-L1 expression (P = 0.0052), and TMB (P = 0.0285) (Fig. 4a–f). In addition, the univariate analyses showed that the NSCLC patients with mutant FGFR4, LUAD, first-line administration of ICIs, ever smoking, elevated PD-L1 score (≥ 50% percentage), or high TMB (≥ 50th percentage) could benefit from ICIs treatment. Based on these variables, we established a nomogram (Fig. 4g). According to this novel nomogram, clinical physicians could easily obtain a point based on each variable, and then evaluate the total point as the sum of all variable points. The efficacy of ICIs treatment could be quantified before treatment for advanced NSCLC patients. In particular, the C-index of this nomogram was 0.766 (95% CI 0.697–0.835), which indicates that the nomogram has a relatively accurate clinical value to predict the PFS of advanced NSCLC patients with ICIs treatment. The calibration plots displayed great predictive performance, which the dashed lines in the calibration plots correspond to a 10% margin of error (Fig. 4h, i). According to the research above, we reasonably deduced that the mutation of FGFR4 can be used as a novel biomarker for ICIs treatment, and could have a connection with factors that are proven to be related to sensitivity to ICIs treatment.

### The mutation of FGFR4 is associated with tumor-infiltrating immune cells in NSCLC

**Fig. 5:**
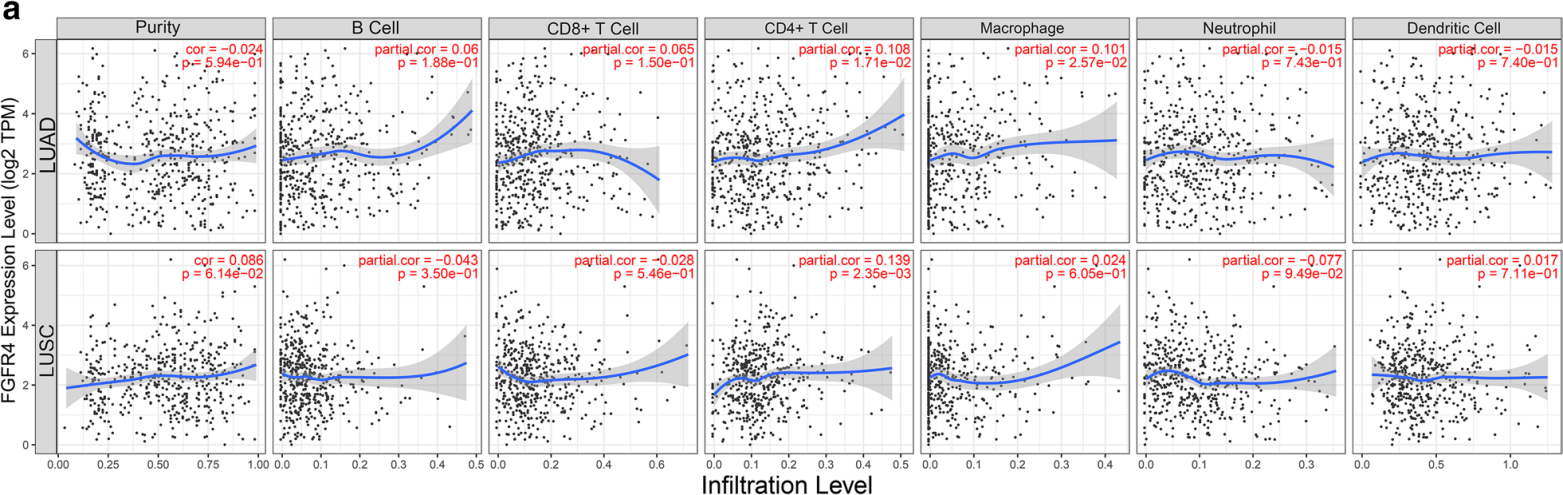
The mutation of FGFR4 are closely associated with the purity of six types TIICs. **a** The correlations between FGFR4 expression and the abundances of B cell, CD8+ T cell, CD4+ T cells, macrophage, neutrophil and dendritic cells in LUAD and LUSC

In order to determine whether FGFR4 effectively reflected the status of the tumor immune microenvironment (TIME), the relationships between the FGFR4 and six TIICs (B cells, CD4 T cells, CD8 T cells, neutrophils, macrophages, and dendritic cells) were analyzed by TIMER online tool. The results showed that the expression of FGFR4 was closely relate with six TIICs (Fig. 5a). Based on the results of TIMER online tool and enrichment analysis above, FGFR4 deficiency might regulate the TIME by activating of the antigen presentation process and cellular immunity to the change in the sensitivity to ICIs treatment in NSCLC.

### Verification of the prognostic significance of FGFR4 in pan-cancer

**Fig. 6:**
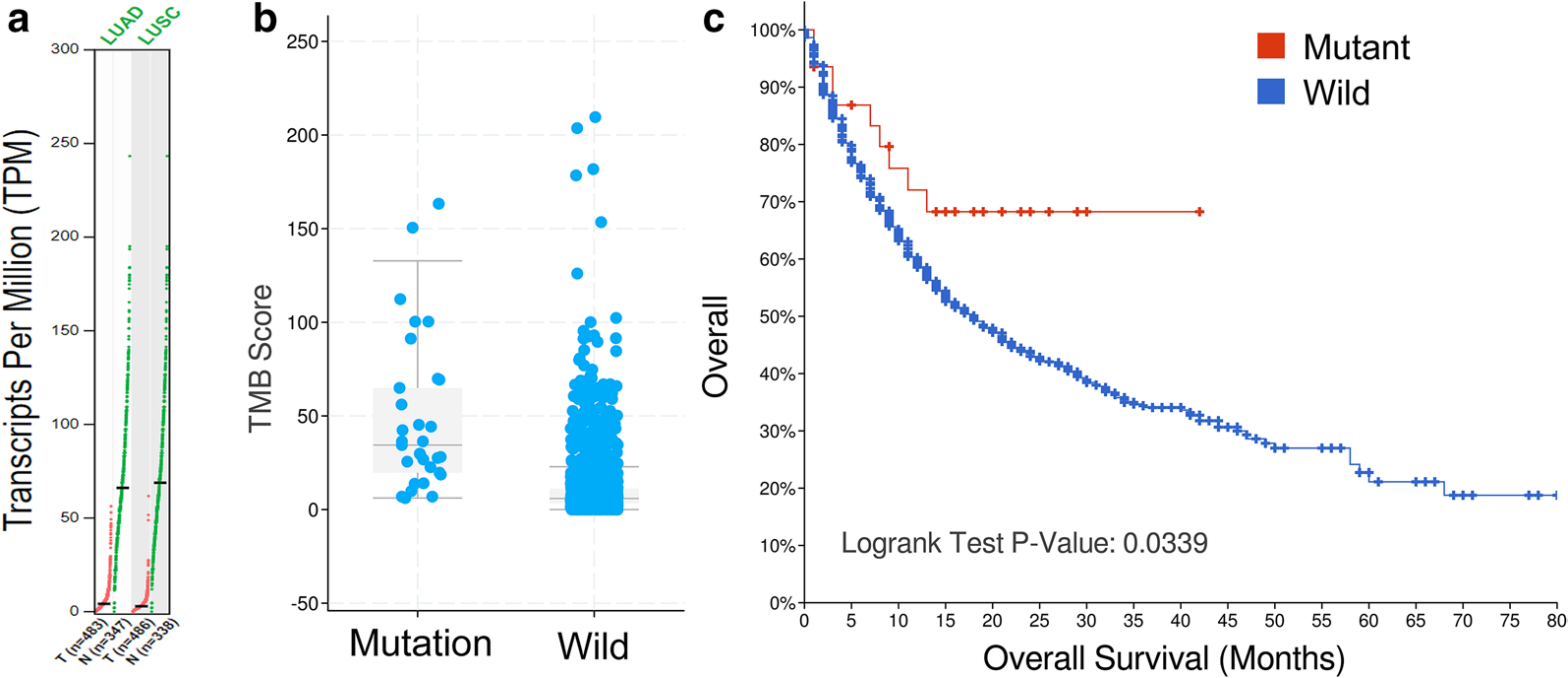
Verification of the prognostic significance of FGFR4 in pan-cancer. **a** The expression of FGFR4 in GEPIA database. **b** The mutation status of FGFR4 is associated with the TMB level. **c** The survival curves based on the mutational status of FGFR4

We analyzed the prognostic value of FGFR4 in another cohort [TMB and Immunotherapy (MSKCC, Nat Genet 2019)] in cBioPortal for Cancer Genomics as an independent external validation. The expression of FGFR4 was validated by GEPIA database (Fig. 6a). The mutation status of FGFR4 were associated with a higher TMB value (P < 0.0001) (Fig. 6b). The Kaplan–Meier survival analysis showed that the expression of FGFR4 was closely related to the prognosis of cancer patients (P-value = 0.0339) (Fig. 6c).

## Discussion

Fibroblast growth factor receptor 4 (FGFR4) is a member of a highly conserved tyrosine kinase family. Previous studies showed that FGFRs is widely associated with many physiological processes, such as embryonic and postnatal development, metabolic homeostasis, and tissue maintenance and repair [19]. Moreover, fibroblast growth factor (FGF) signaling through its receptors, FGFR1, FGFR2, FGFR3, or FGFR4, regulates cell fate, angiogenesis, immunity, and metabolism [20]. Aberrant FGFR activation has been influenced the development and progression of various cancer, such as lung cancer, breast cancer, liver cancer, gastric cancer, uterine cancer, and bladder cancer [21–25]. Overexpression of FGFR4 at the RNA level, overexpression of the FGFR4-specific ligand, and an FGFR4 single nucleotide polymorphism are frequently observed in most tumors [26]. Like other FGFR family members, aberrant FGFR4 activation is closely related to cancer progression and resistance to anti-cancer therapy.

Nowadays, the expression of PD-L1 or TMB level did not show satisfied efficiency in selecting patients who might benefit from ICIs treatment. Skoulidis et al. [27] found that the co-occurring genomic alterations of TP53 and LKB1 serve as a significant role in modulating the tumor microenvironment of NSCLC and related to the sensitivity to ICIs treatment. Sun et al. [28] showed that the mutation of subunits of ARID1 serve as novel biomarkers for the sensitivity to ICIs treatment and prognosis of advanced NSCLC. Based on these studies, we conducted research to further investigate the significance of FGFR4 mutations in predicting the prognosis of advanced NSCLC with ICIs treatment.

In this work, we aimed to analyze the relationship between IRGs and the prognosis of NSCLC patients with ICIs. A total of 274 differentially expressed IRGs were identified, including 170 up-regulated and 104 down-regulated genes. GO and KEGG enrichment analysis showed that these differentially expressed IRGs were mainly associated with leukocyte migration, extracellular matrix, receptor regulator activity and cytokine–cytokine receptor interaction. Only the mutation of FGFR4 have shown to be associated with the prognosis of advanced NSCLC with ICIs treatment in cBioPortal cohort. In addition, the mutation status of FGFR4 is closely associated with other predictive biomarker of ICIs treatment, such as the PD-L1 expression, TMB level and TIICs. Patients harboring FGFR4 mutations might showed elevated of TMB and PD-L1 expression. Moreover, FGFR4 deficiencies could regulate the TIICs and trigger a therapeutic immune response to tumors by the activation of the antigen presentation process and anti-cancer cellular immunity in NSCLC. According to these results, we established a novel nomogram based on the mutation of FGFR4, TMB level, PD-L1 expression, and other clinicopathological parameters for advanced non-small cell lung cancer patients with immune checkpoint inhibitors. This novel nomogram can be used to estimate the survival risks of ICIs treatment, and determine the appropriate treatment plan and follow-up before treatment for NSCLC patients.

Based on existing reports, the mechanism of FGFR4 in advanced NSCLC patients with ICIs treatment is still unclear, which requires follow-up research to further explore. Our research is the first to use large database to establish a nomogram for predicting the prognosis of advanced NSCLC patients with immunotherapy, which undoubtedly provides a new clinical strategy for the ICIs treatment of advanced NSCLC patients. Additionally, our research is the first to illustrate the role of FGFR4 in prognosis of advanced NSCLC with immunotherapy. The function of FGFR4 in the prognosis of NSCLC were clearly clarified. FGFR4 deficiency was closely associated with the poor prognosis of NSCLC patients. The mutation of FGFR4 and the resultant functional deficiencies were closely related to the sensitive phenotype for cancer immunotherapy. We proposed that FGFR4 might be important biomarker in cancer immunotherapy and the prognosis of NSCLC based on our work. However, our research still has certain limitations. First, the clinical sample size of this study is small, and further investigations into the role of FGFR4 in NSCLC with immunotherapy are needed. Then, although this work shows that FGFR4 may participate in the modulation of the TIME and are related to the elevations in TMB and PD-L1 expression. The molecular mechanism underlying these correlations still need to be validated in vivo and in vitro experiments. There are not enough clinical studies to confirm our results.

## Conclusion

To sum up, FGFR4 could serve as a novel biomarker for advanced NSCLC patients with ICIs treatment. The mutation status of FGFR4 is closely associated with other predictive biomarker of ICIs treatment, such as the PD-L1 expression, TMB level and TIICs. Our novel nomogram combines these biomarkers to provide new treatment strategies for non-small cell lung cancer patients with ICIs treatment.

## Supplementary Information

**Additional file 1.** Differentially expressed IRGs of limma results.

**Additional file 2.** Differentially expressed IRGs of edgeR results.

## Data Availability

All data generated during this study are included in this published article. The datasets generated in the current study are available in the cBioportal for Cancer Genomics [29, 30] (http://www.cbioportal.org/).

## Abbreviations

FGFR4: Fibroblast growth factor receptor 4
NSCLC: Non-small cell lung cancer
IRGs: Immune-related genes
MMR: Mismatch repair
MSI-H: Microsatellite instability-high
PD-L1: Programmed death ligand 1
TMB: Tumor mutational burden
ICIs: Immune checkpoint inhibitors
TIICs: Tumor-infiltrating immune cells
TIME: Tumor immune microenvironment
TCGA: The Cancer Genome Atlas
ImmPort: Immunology Database and Analysis Portal
PFS: Progress free survival
GO: Gene ontology
KEGG: The Kyoto Encyclopedia of Genes and Genomes
NGS: Next-generation sequencing
